# A Chitin Deacetylase-Like Protein Is a Predominant Constituent of Tick Peritrophic Membrane That Influences the Persistence of Lyme Disease Pathogens within the Vector

**DOI:** 10.1371/journal.pone.0078376

**Published:** 2013-10-17

**Authors:** Toru Kariu, Alexis Smith, Xiuli Yang, Utpal Pal

**Affiliations:** Department of Veterinary Medicine and Virginia-Maryland Regional College of Veterinary Medicine, University of Maryland College Park, Maryland, United States of America; University of Minnesota, United States of America

## Abstract

*Ixodes scapularis* is the specific arthropod vector for a number of globally prevalent infections, including Lyme disease caused by the bacterium *Borrelia burgdorferi*. A feeding-induced and acellular epithelial barrier, known as the peritrophic membrane (PM) is detectable in *I. scapularis*. However, whether or how the PM influences the persistence of major tick-borne pathogens, such as *B. burgdorferi*, remains largely unknown. Mass spectrometry-based proteome analyses of isolated PM from fed ticks revealed that the membrane contains a few detectable proteins, including a predominant and immunogenic 60 kDa protein with homology to arthropod chitin deacetylase (CDA), herein termed *I. scapularis* CDA-like protein or IsCDA. Although *IsCDA* is primarily expressed in the gut and induced early during tick feeding, its silencing via RNA interference failed to influence either the occurrence of the PM or spirochete persistence, suggesting a redundant role of IsCDA in tick biology and host-pathogen interaction. However, treatment of ticks with antibodies against IsCDA, one of the most predominant protein components of PM, affected *B. burgdorferi* survival, significantly augmenting pathogen levels within ticks but without influencing the levels of total gut bacteria. These studies suggested a preferential role of tick PM in limiting persistence of *B. burgdorferi* within the vector. Further understanding of the mechanisms by which vector components contribute to pathogen survival may help the development of new strategies to interfere with the infection.

## Introduction

In many invertebrates, including blood-feeding arthropods, an acellular strip-like structure, termed peritrophic membrane (PM), occurs within the feeding gut [1]. The membrane can be transient; it is formed during early stages of blood meal engorgement in arthropods and primarily composed of chitin, along with a limited number of accessory proteins, glycoproteins, and glycans. The PM essentially acts as a semipermeable barrier that permits transport of selected materials, such as metabolites and small molecules, between the lumen and surrounding gut tissues. Besides contributing to the compartmentalization of the gut and playing a vital role in physiology, the PM also acts as an effective mechanical barrier, preventing transport of small cell organelles, such as ribosomes, which could be derived from an ingested blood meal and retained in the endoperitrophic luminal spaces [2]. The PM has been shown to influence the persistence of a select set of pathogens within blood-sucking arthropods, especially ones that colonize or traverse the gut epithelial tissue. In *Aedes aegypti* mosquitoes, PM formation coincides with the onset of blood meal engorgement and rapidly ends within 12 hours [3]. On the other hand, to thwart this mechanical barrier and traverse the PM, the *Plasmodium* parasite is known to secrete chitinases, which enable its movement from the luminal compartment to the epithelial cells [4]. Similarly, *Leishmania* parasites secrete a chitinase that also helps pathogen dispersal through the sand fly gut [5]. Several reports described the existence of a solid PM in *Ixodes* ticks that is clearly visible within 18-36 hours of the onset of feeding [6]. Notably, *Babesia microti*, which is transmitted by *Ixodes* ticks, is known to possess a highly specialized organelle, called an arrowhead, which facilitates pathogen dispersal through the PM [2]. Despite these studies, information on the molecular organization of the PM in *I. scapularis* - the principal arthropod vector for Lyme disease and many other human infections - is limited. It is also unknown whether or how this mechanical barrier influences the persistence or transmission of prevalent tick-borne pathogens, such as *B. burgdorferi*, which can replicate within the gut lumen of a feeding *I. scapularis*, colonize gut tissues, or invade the gut epithelia [7]. As the PM is also thought to play important yet undefined role(s) in gut physiology [1], further studies on its structure, properties, and function will enrich our understanding of vector biology and help combat arthropod-borne infections.

Studies on model insects revealed that the molecular or biochemical constituents of the PM vary considerably amongst arthropods. Although chitin constitutes a major component of the PM in most species, in certain species, its presence is insignificant [8]. This sugar is a polymer of *N*-acetyl-D-glucosamine with a β-1,4 linkage that is structurally analogous to that in cellulose. In fact, the PM is organized into a highly structured lattice of chitin fibrils held together by chitin binding proteins, while glycans fill the interstitial spaces. In most cases, proteins that are associated via ionic or covalent interactions constitute the major component of the PM [1]. However, the number of unique proteins in the PM of a given species varies greatly, from only a few to several dozen. These proteins are often classified as different types based on how they can be released from the matrix [9]. While the members of one class are covalently linked and thus cannot be separated, proteins of other classes are removable to a certain degree by a simple saline wash or can be extracted with mild to strong denaturants. The latter classes of proteins are also known as peritrophins, which commonly contain chitin-binding domains for specific protein-PM interaction [9] in addition to mucin-like domains that are rich in serine, threonine, and proline [10]. In some cases, peritrophins employ a chitin deacetylase (CDA)-like domain for chitin binding [10]. CDAs are metalloproteins that catalyze the release of acetyl groups from chitin [11]; although their enzymatic functions are well characterized in microbes, their role(s) in arthropod biology remain unknown [12]. In this report, we describe our efforts to identify the molecular constituents of the *I. scapularis* PM and their roles influencing the persistence of Lyme disease pathogens within the vector.

## Materials and Methods

### Ethics statement

Animals were treated in compliance with the Guide for the Care and Use of Laboratory Animals. All animal experiments were performed in accordance with the guidelines and approval of the Institutional Biosafety Committee and the Institutional Animal Care and Use Committee at the University of Maryland, College Park. 

### Mice, bacterial strain, and ticks

Five-week-old female C3H/HeN mice were purchased from the National Institutes of Health. The *B. burgdorferi* isolate A3 is a low-passage, infectious, and clonal derivative of the type strain B31, which was used for this study. *Borrelia* cultures were grown in Barbour-Stoenner-Kelly H (BSK-H) media supplemented with 6% rabbit serum at 34°C. *Ixodes scapularis* ticks used in the present study were reared in the laboratory as described elsewhere [13].

### Isolation of peritrophic membrane (PM)

We found that the PM can be more easily isolated from post-fed ticks. Nymphal *I. scapularis* ticks were allowed to engorge on mice (3 animals, 25 ticks/mouse) as described [13], and replete ticks were dissected within a drop of sterile distilled water under a zoom stereomicroscope (Olympus) equipped with 2X auxiliary objective lens (total magnification 90x) and a bright fiber optics light source. The isolated gut diverticula were transferred to a new drop of water, carefully pinched into smaller fragments with forceps, and further washed by repeated transfer into new drops of water. During this washing process, the brownish materials (gut cells and blood meal) slowly disintegrate and disappear, and the PM is finally visible as an insoluble, transparent, film-like structure. To assess the purity, the isolated PM materials were subsequently labeled with WGA-FITC and propidium iodide and processed for confocal microscopy. 

### Protein analysis and identification via liquid chromatography-mass spectrometry (LC-MS/MS)

For the identification of constituent proteins, PM structures isolated from 5-10 ticks were dissolved in SDS-PAGE sample buffer and separated in a Mini-PROTEAN® gel electrophoresis system (Bio-Rad laboratories) using 12% SDS-PAGE gels. Three clearly separated and visible gel bands were excised, independently subjected to tryptic in-gel digestion, and finally processed for LC-MS/MS-based protein identification at the university core facility as detailed in our earlier publications [14,15]. The LC-MS/MS data files were analyzed using two search engines: Sequest via Bioworks (Thermo Electron) and Mascot via an in-house Mascot Server (Matrix Science). Results were combined using Scaffold Distiller (Proteome Software) for the identification of proteins. 

### Protein expression, polyclonal antibody preparation, and Western blotting

Recombinant IsCDA was produced in *Escherichia coli* using the bacterial expression vector pGEX-6P-1 (GE Healthcare) with specific primers, 5’-ATT GAA TTC GCC TCA TTG CCG CCC GTC CT- 3’ and 5’-ATT CTC GAG TCA CCT GTA GAG CTT GGT GTC GA -3’. Expression, purification, and enzymatic cleavage of the glutathione transferase (GST) fusion proteins were carried out as detailed [13]. For the generation of polyclonal antisera, IsCDA (without a fusion partner) produced in *E. coli* (10 µg/animal) or isolated native PM (dissected from 40 fed nymphal *I. scapularis* ticks) was emulsified in complete Freund's adjuvant and injected into groups of mice. The animals were boosted two times at 14-day intervals with the same dose of antigen in incomplete Freund's adjuvant, and sera were collected one week following the second boost. Western blotting was performed as detailed in our earlier publication [16]. 

### Microscopy

Transmission electron microscopy was performed as described [13] with the following minor modifications. Briefly, dissected guts from naïve unfed or 36 h fed nymphal *I. scapularis* were fixed with 2.5% glutaraldehyde in 0.1 M sodium cacodylate buffer (pH 7.4) followed by 1% osmium tetroxide in 0.1 M sodium cacodylate buffer. Grids were then embedded in 0.67% uranyl acetate in 1.8% methylcellulose and examined on a FEI Tecnai 12 BioTWIN transmission electron microscope. 

For confocal microscopy, tissues from engorged ticks were fixed for 24 h at 4°C in 4% (w/v) paraformaldehyde in PBS and then embedded in a Tissue-Tek (Sakura Finetek). Cryosectioning was performed at -20°C using a cryotome, and 10 μm thick sections were incubated with 10 µg/ml FITC-labeled lectin (WGA-FITC, Sigma). Slides were then washed using PBS with 0.05% Tween 20, stained with propidium iodide (PI), and imaged by a LSM 510 laser confocal microscope (Zeiss) as described [17]. Isolated PM samples were also similarly processed for confocal microscopy or directly observed under a bright field microscope. Production of IsCDA in the fed tick gut was also analyzed by immunofluorescence microscopy using polyclonal antisera against recombinant IsCDA and Alexa 488-labeled secondary antibodies. Tick nuclei were stained with DAPI.

### Infection studies and measurement of pathogens

For studies involving *B. burgdorferi* acquisition by ticks, pathogens (10^5^ cells/mouse) were introduced into mice by syringe inoculation. At two weeks following inoculation, groups of naïve ticks (2 mice/group, 20 ticks/mouse) were allowed to parasitize infected mice, and replete ticks were collected for measurement of borrelial burdens by quantitative RT-PCR (qRT-PCR) analyses. For studies involving pathogen transmission from the vector, nymphal ticks naturally infected with *B. burgdorferi* were generated [14] and allowed to parasitize naïve C3H mice (3 mice/group, 5 ticks/mouse). Engorged ticks were collected and individually processed for qRT-PCR analyses to determine pathogen burdens in the vector. DNA-free RNA samples were isolated from ticks at various times following feeding, and qRT-PCR analyses were performed as described [18]. For assessment of *B. burgdorferi* levels in tissues, *flaB* transcripts were measured and then normalized to the level of tick *β-actin* transcripts [17]. 

### RNA interference

An RNA interference (RNAi) study was performed as detailed earlier [19]. Briefly, cDNA from nymphs was prepared, and four separate regions of *IsCDA* were amplified using the following sets of primers containing *Sac*I and *Kpn*I restriction sites: dsR1 5'- AAT GAG CTC AAG AGA TAT CTG GG -3' and 5'- AAT GGT ACC AGT CCT CCC GGT GGC AGT TC -3'; dsR2 5'- AAT GAG CTC CGA CGT CGA TTA CAA GCT CA -3' and 5'- AAT GGT ACC ACG GCA TCT TGA ATC CGT AG -3'. Notably, as the *IsCDA* ORF contains native *Sac*I and *Kpn*I sites in the nucleotide position 385 and 1192, respectively, a longer fragment (corresponding to the position between 55 and 1623) was amplified using the primers 5’-ATT GAA TTC GCC TCA TTG CCG CCC GTC CT- 3’ and 5'- AAG AGC TCT CAA TGT CCA AGA GGA TTC T-3', and two additional constructs - (dsR3, position 385-1192 and dsR4, position 1192-1623) - were generated using further digestion with *Sac*I and *Kpn*I. A 400 bp *GFP* fragment (Clontech) was also amplified using the following primers: 5'- AAT GAG CTC GAG GTG AAG TTC GAG GGC GA -3' and 5'- AAT GGT ACC TCC ATG CCG AGA GTG ATC CC -3'. The *IsCDA* and *GFP* amplicons were separately cloned into the corresponding restriction sites of the L4440 double T7 script vector [19], and 30-100 µg of dsRNA were synthesized in a single reaction using a commercial kit (MEGAscript RNAi kit, Invitrogen). The purity of the RNA preparation was analyzed by agarose gel electrophoresis as well as absorbance measurements at 260 and 280 nm using a NanoDrop spectrophotometer. Five μl of the dsRNA (10 μg/μl) was loaded into a capillary tube and rectally microinjected into the gut of unfed nymphs (50 ticks/group) as described [20,21]. The injected nymphs were used for feeding on mice at 3 h after injection, and individual replete ticks were processed for assessment of gene silencing as well as pathogen levels using qRT-PCR analysis. Primers that bind further upstream and downstream of the *IsCDA* cDNA encompassing the dsRNA sequence (5'- TCT AAG GTT CGC TGC CTC AT -3' and 5'- TCT CCT TCT GCG ATG TGT TG -3') were used for the qRT-PCR. 

### Antibody transfer studies

 Anti-IsCDA antibodies or normal mouse serum was microinjected into naïve or infected nymphs as described earlier [22]. At 1 h after injection, the ticks were allowed to feed on naïve (acquisition) or infected (transmission) mice. Levels of *B. burgdorferi* and tick commensal bacteria were determined by qRT-PCR using primers for borrelial *flab* and 16S rRNA (5’-TCC TAC GGG AGG CAG CAG T-3’ and 5’-GGA CTA CCA GGG TAT CTA ATC CTG TT-3’).

### Bioinformatics and statistical analyses

Protein annotation and searches were executed using the NCBI (www.ncbi.nlm.nih.gov) and VectorBase (www.vectorbase.org) databases. Analyses of protein families were performed using the Pfam database (http://pfam.sanger.ac.uk/). *In silico* analyses for determining putative signal peptides of PM proteins were executed by the SignalP 3.0 server (www.cbs.dtu.dk/services/SignalP). For prediction of protein homology, amino acid sequences were aligned using the GENETYX version 6 software (GENETYX Corporation). 

Results are expressed as the mean ± standard error (SEM). The significance of the difference between the mean values of groups was evaluated by two-tailed Student’s *t*-test.

## Results

### Identification and isolation of *I. scapularis* PM

**Figure 1:**
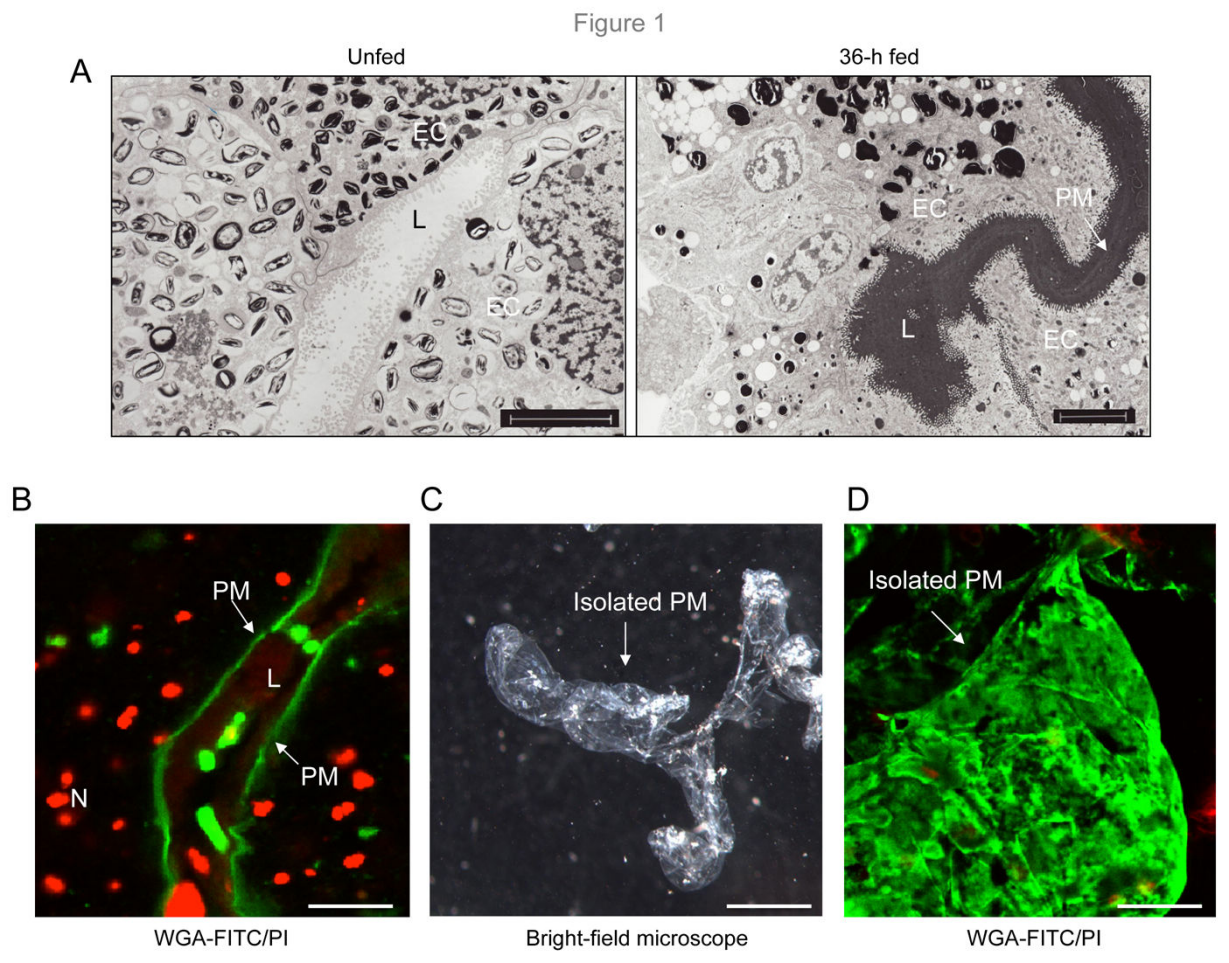
Analysis and isolation of peritrophic membrane in *I. scapularis*. (A) Transmission electron microscopic detection of peritrophic membrane (PM). While undetectable in the gut of unfed ticks (left panel), a PM structure is conspicuous in ticks and was isolated and processed at 36 h of feeding on mice (L, lumen; EC, gut epithelial cell). Arrows point to the PM. Scale bar, 5 μm. (B) Immunofluorescence localization of PM. Cryosections from unfixed tick gut samples were labeled with WGA-FITC (green) and propidium iodide (PI, red) and imaged using a confocal microscope. Arrows point to the green PM, while the nuclei (N) of gut epithelial cells are labeled red. Scale bar, 10 μm. (C) Isolation of an intact PM structure. The PM was isolated from the dissected gut of nymphal ticks and viewed under a binocular dissecting microscope. Scale bar, 100 μm. (D) Relative purity of the extracted PM. The PM, as shown in Figure 1C, was stained with WGA-FITC (green) and PI (red) and imaged using a confocal microscope. Lack of red fluorescence indicated absence of contaminating gut cells. Scale bar, 20 μm.

In agreement with previous studies [6], a conspicuous PM-like structure is readily recognizable in ticks using transmission electron microscopy (Figure 1A). We next sought to determine that the structure is also detectable by light fluorescence microscopy using FITC-tagged wheat germ agglutinin (WGA) - a lectin that binds to *N*-acetylglucosamine-containing substances (Figure 1B). To characterize the PM further, we attempted to isolate the structure using a high-powered dissecting binocular microscope. To accomplish this, fed ticks were dissected under a few drops of sterile water, and the gut was isolated. The organ was thoroughly washed until the tissues were disintegrated, exposing the acellular and transparent PM structure (Figure 1C), which was carefully separated. The PM preparations were further washed repeatedly with sterile distilled water to remove any traces of contaminating gut cells and blood meal contents, as confirmed by subsequent labeling with FITC-WGA and propidium iodide (Figure 1D). 

### Identification of major antigenic components of PM

**Figure 2:**
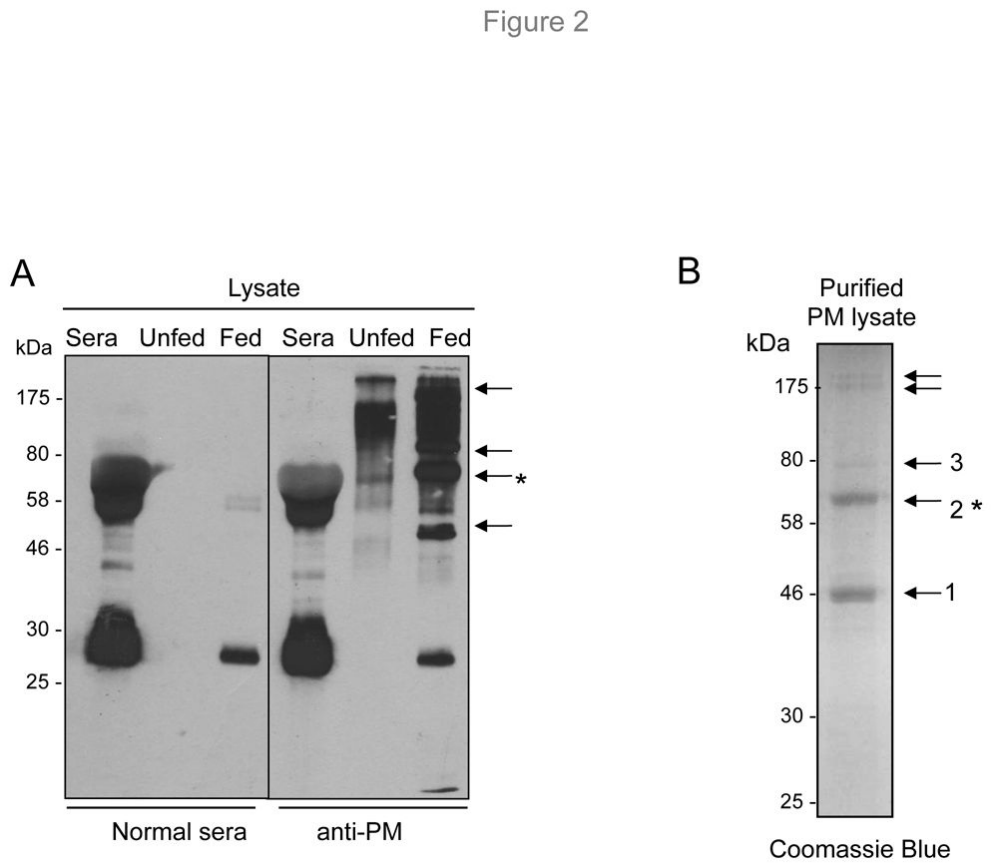
Identification of tick PM proteins. (A) Western blot analysis of PM proteins from the tick gut. Gut samples dissected from unfed and fed ticks were immunoblotted using antibodies generated against the PM. Specificity of anti-PM antisera was tested against normal mouse serum (present in fed ticks) or by replacing the primary antibody with normal mouse serum. Arrows denote proteins (between 46-175 kDa) that are preferentially recognized by the anti-PM antibodies, suggesting specific association with the PM in the fed vector gut. (B) Identification of abundant PM proteins. PM samples were isolated from fed ticks and extensively rinsed with water. Proteins in the solubilized PM fraction were separated by SDS-PAGE and stained with Coomassie Brilliant Blue. Clearly resolved and detectable protein bands, as indicated by arrows (1-3), were excised and analyzed by LC-MS/MS spectrometry for identification of the proteins. Migration of the protein MW marker is indicated on the left (in kDa). Asterisks indicate the IsCDA protein that is the focus of the present work.

Our initial attempts to identify antigenic components of purified *I. scapularis* PM using a panel of polyclonal antibodies raised against isolated mosquito PM structures (kindly provided by Dr. Marcelo Jacobs-Lorena) were unsuccessful. This suggests that the major immunogenic components of the tick PM likely differ from their mosquito counterparts. We therefore collected purified PM from 120 fed nymphal ticks and generated specific murine antisera by immunizing murine hosts. Generated polyclonal antibodies were then used to detect antigenic components of the tick PM. The results show that while the antibodies reacted with multiple proteins in the unfed nymphal tick, there is a smear of high-molecular-weight proteins in fed ticks typical of glycosylated antigens; at least four PM-specific antigens that migrated with molecular masses around 46, 60, 80, and 175 kDa were conspicuous (Figure 2A). To identify the proteins associated with the PM, the structure isolated from ticks was dissolved in SDS-PAGE sample buffer and resolved in a preparative gel. At least three visible bands were clearly resolved, excised, and subjected to LC-MS/MS analyses (Figure 2B), which identified three major PM-associated proteins (Table 1). While database searches indicated that two of these proteins (ISCW14049 and ISCW19094) lack specific homology to proteins of known function, the third member (ISCW000083) represented a protein with a conserved enzymatic domain (polysaccharide deacetylase, Pfam entry PF01522) with significant homology to arthropod chitin deacetylase. ISCW000083 is therefore referred to herein as *I. scapularis* CDA or IsCDA. As we have determined that the protein is highly immunogenic (asterisks, Figure 2) and orthologs are known to be important for PM biology and can be modulated during arthropod infection [23], IsCDA constituted the focus of our further studies.

**Table 1:** Three abundant proteins identified by LC-MS/MS analysis from the *I. scapularis* gut PM.

**Band**	**VectorBase accession number**	**NCBI accession number**	**Molecular Weight (predicted)**	**Annotation (NCBI/VectorBase)**	**Domain (pfam)**
1	ISCW014049	EEC19801	32 kDa	Peritrophic membrane chitin binding protein, putative	Unknown
2	ISCW000083	EEC01339	60 kDa	Peritrophic membrane chitin binding protein, putative	Polysaccharide deacetylase (PF01522)
3	ISCW019094	EEC08688	40 kDa	Hypothetical protein	Unknown

### Characterization of *I. scapularis* CDA (IsCDA)

**Figure 3:**
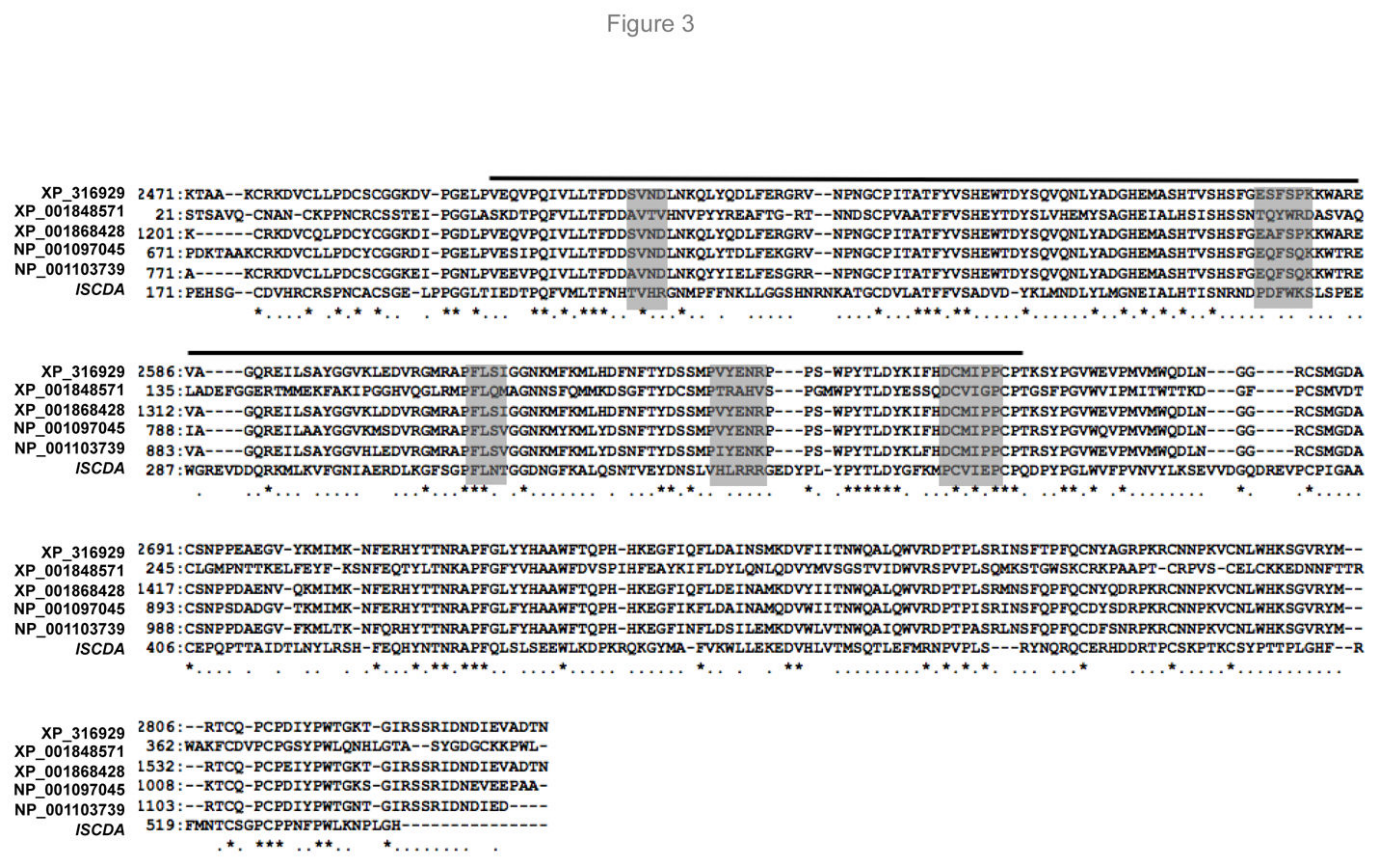
Amino acid sequence alignment of potential catalytic domains of CDA with orthologs from other insect species. Partial amino acid sequences (annotations are shown according to NCBI reference sequences) from *Anopheles gambiae* (XP_316929), *Culex quinquefasciatus* (XP_001848571 and XP_001868428), *Drosophila melanogaster* (NP_001097045), and *Tribolium castaneum* (NP_001103739) were aligned with *IsCDA* using the GENETYX software. The chitin-binding and catalytic domains are indicated with dark lines, while CDA motifs are denoted by gray boxes.

**Figure 4:**
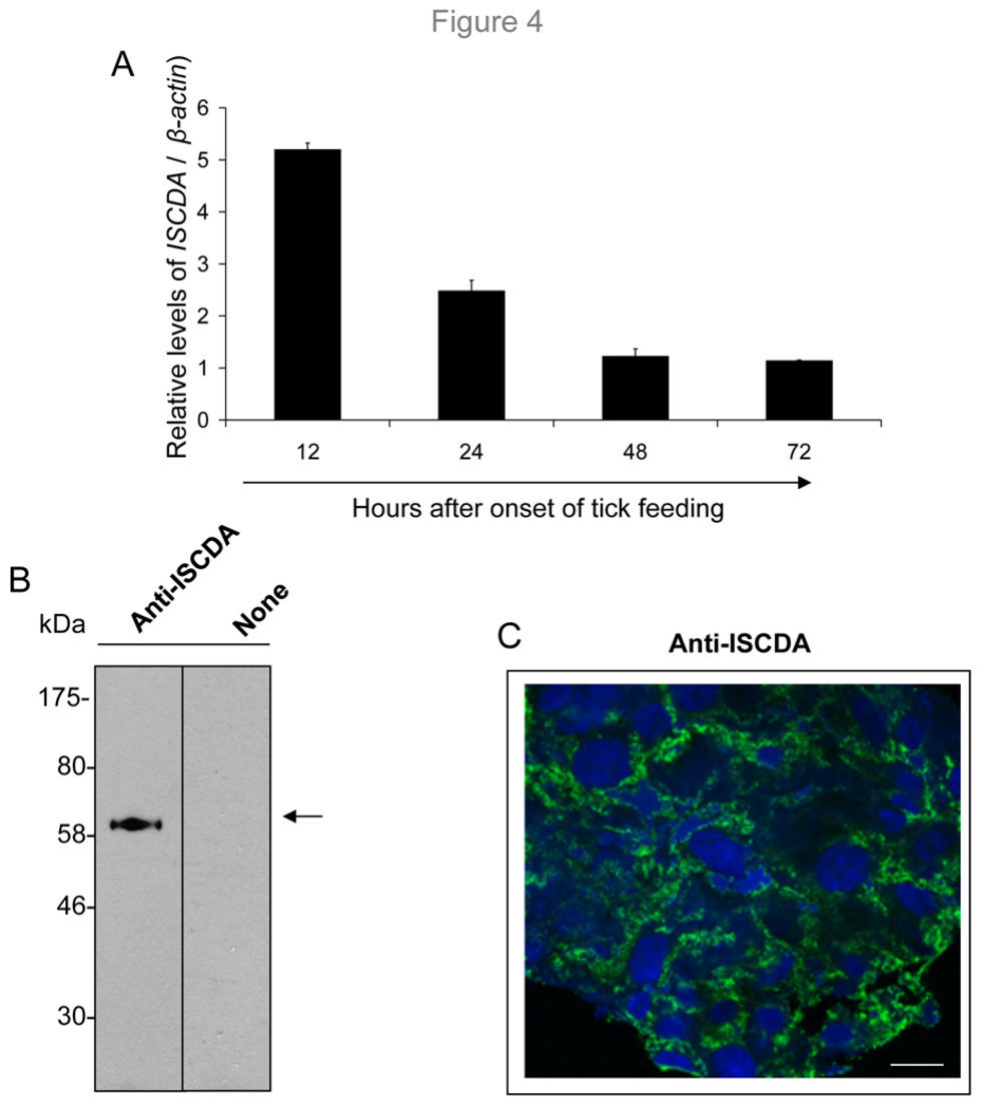
Temporal and spatial distribution of IsCDA in tick gut. (A) The highest levels of *IsCDA* expression are noted during early tick attachment on the host. Ticks were collected at various times of attachment to the murine hosts, and *IsCDA* expression was measured using quantitative RT-PCR. (B) Antisera generated against recombinant IsCDA detect a native protein in the PM. The PM structure from the tick gut was isolated and immunoblotted with IsCDA antibodies, which recognized native IsCDA with an approximate MW of 60 kDa (arrow). Migration of the protein MW marker is indicated on the left (in kDa). (C) Cellular localization of IsCDA in the tick gut tissue. Cryosections from unfixed tick gut collected at 24 h of feeding were labeled with antibodies against IsCDA and Alexa 488-labeled secondary antibodies and processed for confocal immunofluorescence microscopy. The tick tissues were labeled with a nuclear stain (DAPI). Scale bar, 10 μm.

Chitin deacetylase (CDA; E.C. 3.5.1.41) is an enzyme that catalyzes the hydrolysis of acetamido groups of *N*-acetylglucosamine in chitin, promoting the conversion to chitosan, a glucosamine polymer. The deduced amino acid sequence of IsCDA contains a putative signal peptide, and the protein displays significant homology to several arthropod CDAs, particularly to orthologs in *Anopheles*, *Culex*, *Drosophila*, and *Tribolium* sp. (Figure 3). In addition to the strong conservation of all cysteine residues, at least five motifs that encompass the active site domain of deacetylase family proteins are also conserved in IsCDA (Figure 3). Quantitative analyses of *IsCDA* transcripts in ticks further indicated that the gene is dramatically expressed during early nymphal feeding (Figure 4A), coinciding with the formation of the PM structure in feeding ticks (Figure 1A). To characterize IsCDA further, we expressed a truncated form of the protein in *E. coli* and generated polyclonal antisera in mice. Immunoblotting assays demonstrated that antibodies generated against recombinant IsCDA recognize a 60 kDa native protein in the PM (Figure 4B). Next, confocal immunofluorescence studies using IsCDA antibodies show that the protein is detectable in the gut tissue (Figure 4C). 

### Knockdown of CDA failed to interfere with PM formation and spirochete persistence

**Figure 5:**
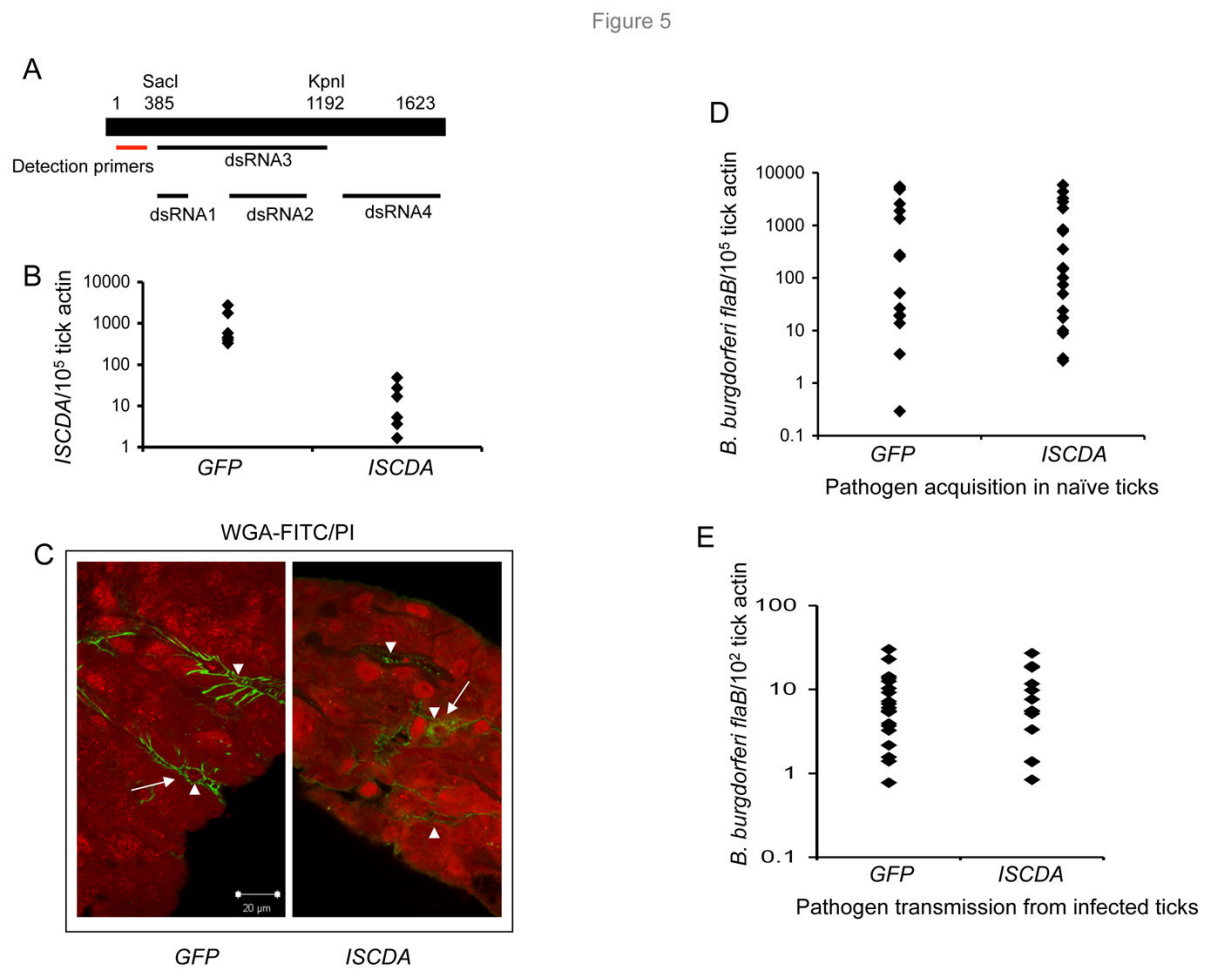
*IsCDA* knockdown failed to interfere with formation of the PM or the persistence of *B. burgdorferi* within *I. scapularis*. (A) Schematic representation of *IsCDA* open reading frame showing regions targeted for RNA interference studies. Out of the four dsRNA constructs generated and tested (relative positions are indicated by straight black lines), dsRNA3 (spanning nucleotide positions 385-1192) was most effective and used for subsequent studies. Location of primers that were used to assess the efficacy of *IsCDA* knockdown via injection of dsRNA3 is indicated by the red line. (B) Knockdown of *IsCDA* transcripts induced by RNA interference. Nymphal ticks (10/group) were injected with *IsCDA* dsRNA or control *GFP* dsRNA and fed on naïve mice to repletion, and isolated guts were processed for quantitative RT-PCR using primers against *IsCDA* and the results normalized against tick *β-actin*. Each diamond represents an individual tick that was processed and analyzed separately. *IsCDA* transcripts levels in *IsCDA* dsRNA-injected ticks were reduced by 1000-fold or more compared to *GFP*-injected control ticks (p <0.05). (C) Injection of *IsCDA* dsRNA failed to influence the formation of the PM in fed ticks. Guts from fed nymphal ticks were cryosectioned, labeled with WGA-FITC (green) and PI (red), as described in Figure 1B, and imaged using a confocal microscope. Note that for a global and comparative assessment of the PM in the gut diverticula of *IsCDA*-knockdown or control ticks, the gut samples were imaged under lower magnification, which renders the PM, appearing as green fluorescent lines (arrows), and luminal spaces (arrowheads), relatively inconspicuous and intermittent across the cryosectioned gut diverticulum. Scale bar, 20 μm. (D) *IsCDA* knockdown failed to interfere with persistence of spirochetes in fed ticks during their acquisition from infected mice. Mice were infected with *B. burgdorferi* and parasitized by naïve nymphal *I. scapularis* that were injected with either *IsCDA* dsRNA or control *GFP* dsRNA. Replete ticks were collected, and *B. burgdorferi* levels were determined using quantitative RT-PCR targeting *flaB* and normalized against tick *β-actin* levels. The difference in spirochete number between the *IsCDA* dsRNA-treated and *GFP* dsRNA-treated groups was nonsignificant, p > 0.05. (E) *IsCDA* knockdown did not influence persistence of *B. burgdorferi* in feeding ticks during pathogen migration to naïve hosts. *IsCDA* dsRNA or control *GFP* dsRNA-injected spirochete-infected ticks were allowed to engorge on naïve mice, and *B. burgdorferi* levels were determined in replete ticks, as detailed in panel C. The difference in pathogen level between the *IsCDA* dsRNA-treated and *GFP* dsRNA-treated groups was nonsignificant, p >0.05.

As IsCDA constitutes an abundant component of the PM, we sought to determine whether RNAi-mediated knockdown of *IsCDA* affects the formation of the PM structure and influences persistence of *B. burgdorferi* within feeding ticks. As the open reading frame (ORF) of *IsCDA* is relatively large (1623 bp), we decided to first identify the specific region that is most susceptible to RNAi-mediated knockdown of *IsCDA* transcripts. To accomplish this, four different dsRNA preparations were generated targeting various regions of *IsCDA* (Figure 5A). As a control for the RNAi experiments, *GFP* dsRNA (400 bp) was also synthesized. Unfed nymphs (20 ticks/group) were microinjected with a dsRNA preparation, allowed to rest for 3 h, and subsequently placed onto naïve mice. After repletion, the nymphs were harvested and assessed for *β-actin* or *IsCDA* transcript levels (using primers that bind *IsCDA* outside of the dsRNA target sequence) via qRT-PCR analyses. The data indicated that compared to the control (*GFP* dsRNA), or other *IsCDA* dsRNA constructs (data not shown), the most significant reduction of *IsCDA* transcripts resulted in the case of dsRNA3 (Figure 5A and 5B), which was used for all subsequent studies. We next assessed whether dsRNA3-mediated down-regulation of *IsCDA* influences the formation of the PM and *B. burgdorferi* persistence. To accomplish this, groups of *IsCDA* knockdown ticks were analyzed for the existence of a PM by confocal immunofluorescence microscopy, and borrelial burdens were assessed using qRT-PCR analyses. The results show that PM formation in ticks that fed on mice infected with *B. burgdorferi* for two weeks is unaffected by RNAi-mediated reduction of *IsCDA* transcripts (Figure 5C). The *B. burgdorferi* levels in these ticks, either during pathogen acquisition from infected mice (Figure 5D) or their migration from the vector to naïve hosts (Figure 5E), are also similar to those of control (*GFP*-injected) ticks. All groups of mice became infected with *B. burgdorferi* at similar levels (data not shown), suggesting that RNAi-mediated knockdown of *IsCDA* failed to influence pathogen transmission to hosts as well.

### IsCDA antibodies interfere with borrelial persistence in feeding ticks during their transitions between vector and murine hosts

**Figure 6:**
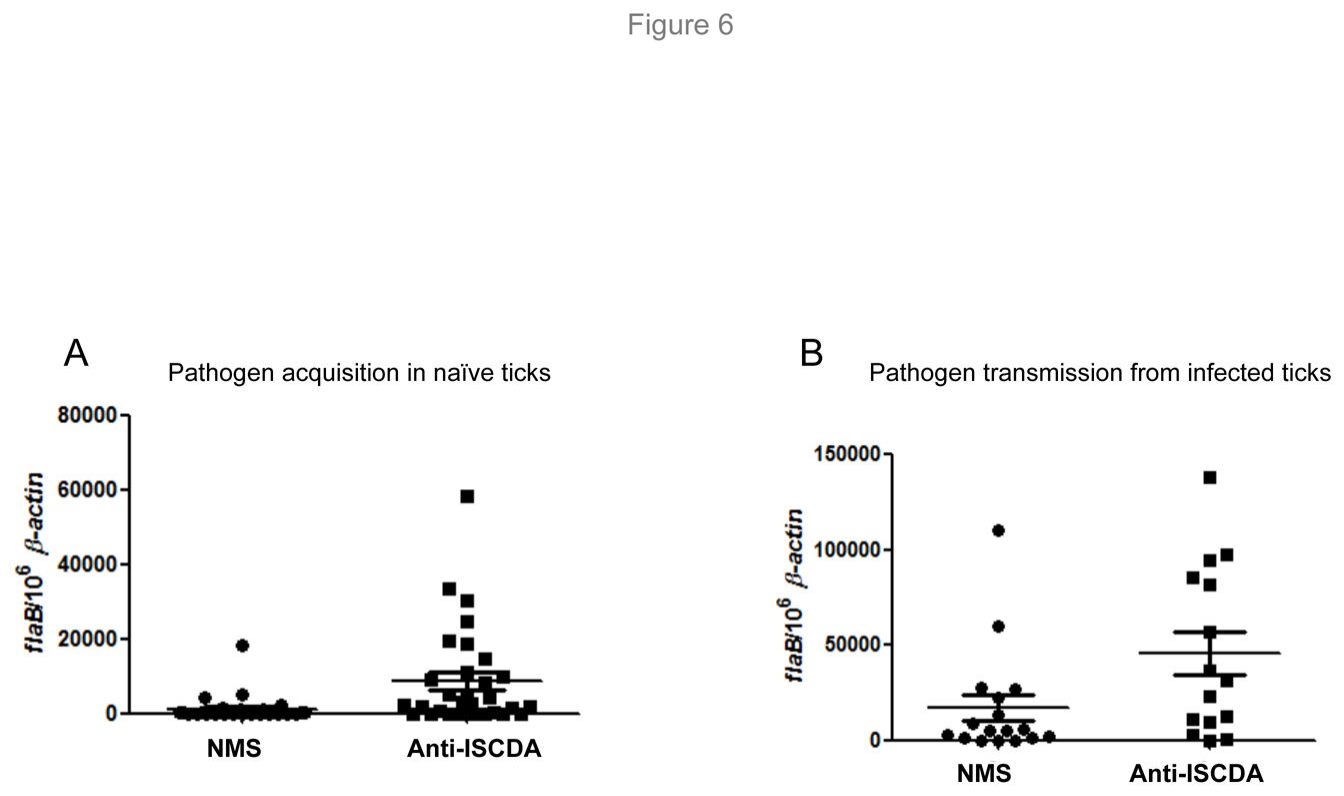
IsCDA antibodies influence the persistence of *B. burgdorferi* in feeding ticks. (A) Passive transfer of antibodies raised against IsCDA interferes with spirochete persistence in fed ticks during their acquisition from infected hosts. Naïve nymphal ticks were microinjected with equal amounts of antibodies against IsCDA or control (normal mouse serum, NMS). *B. burgdorferi* burdens in replete ticks were assessed by qRT-PCR analyses by measuring copies of *B. burgdorferi* *flaB* RNA and normalized against tick *β-actin* levels. Each diamond represents an individual tick that was processed and analyzed separately. The difference in spirochete number between ticks injected with anti-IsCDA antibodies and control (NMS) was significant, p < 0.01. (B) Passive transfer of IsCDA antibodies influences persistence of *B. burgdorferi* in feeding ticks during pathogen transmission to naïve hosts. Spirochete-infected nymphal ticks were microinjected with antibodies, and *B. burgdorferi* burdens in replete ticks were assessed, as detailed in panel A. Each diamond represents an individual tick that was processed and analyzed separately. The difference in spirochete number between ticks injected with anti-IsCDA antibodies and control (NMS) was significant, p < 0.05.

Although the above studies suggested a redundant role of IsCDA in the occurrence of the PM and pathogen persistence, this antigen constitutes an abundant and immunogenic component of the PM. We therefore hypothesized that antibodies against IsCDA should bind the PM, thereby interfering with its normal organization and/or functions, such as altering the porosity of the matrix, inhibiting possible CDA enzymatic activity, or influencing the function of other important PM components via steric hindrance. To explore this, we studied whether passive transfer of antibodies against IsCDA into the tick gut lumen affects *B. burgdorferi* persistence in feeding ticks. To examine such effects on borrelial survival during pathogen acquisition from infected mice, groups of naïve ticks were microinjected with normal mouse serum (NMS) or IsCDA antiserum and allowed to feed on *B. burgdorferi*-infected mice. While treatment with IsCDA antiserum did not alter general gut morphology (data not shown), measuring pathogen levels by qRT-PCR analyses showed that spirochete burdens in replete ticks injected with IsCDA antibodies increased significantly, at least 10-fold compared with those in NMS-injected ticks (Figure 6A). Notably, such effects of IsCDA antibodies on the persistence of total bacterial populations in the gut were unnoticeable, as the level of eubacterial 16S rRNA in IsCDA antiserum-injected ticks remained unaltered compared to that in control ticks (data not shown). Similarly, the IsCDA antibody-mediated effects on borrelial persistence were also noted during pathogen transmission from infected ticks to naïve hosts. Infected ticks injected with IsCDA reflected a significant increase in *B. burgdorferi* levels in the feeding vector compared to control (NMS-injected) ticks, whereas total gut bacterial levels remained unaltered (data not shown). Overall, these studies indicated that IsCDA antibody-mediated interference of PM structure and/or function predominantly influences *B. burgdorferi* persistence in feeding ticks.

## Discussion

We sought to identify major antigenic components of the *I. scapularis* PM in order to assess if genetic or immunological inhibition of the key matrix components interferes with pathogen survival in the vector. We show that the PM proteome possibly contains a limited quantity of unique proteins and that the tick gene product ISCW000083, a protein with a chitin deacetylase (CDA) like-domain, herein termed IsCDA, represents one of the major immunogenic components of the tick PM and was copiously expressed by the gut tissue. Although predominant expression of *IsCDA* during early tick feeding suggested its role in the formation of the PM, RNAi-mediated knockdown of IsCDA showed its redundant roles in PM generation and *B. burgdorferi* persistence in ticks. Nevertheless, passive transfer of anti-IsCDA antibodies specifically enhanced spirochete persistence in fed ticks, suggesting a potential role of the PM in influencing pathogen persistence in the vector. However, our data cannot explain how the PM limits *Borrelia burgdorferi* persistence within ticks or precisely how IsCDA antibodies modify the PM or influence the survival of spirochetes, which remain interesting subjects of future investigation. 

Arthropod CDAs are secreted metalloproteins that catalyze the release of acetyl groups from chitin, a homopolymer of β-(1,4)-linked *N*-acetylglucosamine (GlcNAc), to form chitosan, a homopolymer of β-(1,4)-linked D-glucosamine [24]. Although the precise molecular functions of IsCDA in PM or gut biology remain to be determined, the protein features a signal peptide as well as five catalytic motifs potentially encompassing the active site of the deacetylase domain, suggesting a potential enzymatic role of IsCDA in PM modification. Of note, the roles that insect CDAs play in arthropod physiology are not well understood, although they are thought to modify PM structure and organization by deacetylating *N*-acetyl-D-glucosamine. In *D. melanogaster*, two CDA-like proteins, CDA1 (serpentine) and CDA2 (vermiform), were thought to be involved in the deacetylation of the terminal *N*-acetyl-D-glucosamine, which is extended to form the chitin chain [25]. As deacetylation increases the solubility and decreases the density of chitin fibrils [26,27], influencing their structure and orientation, CDA function can alter the physical and chemical properties of the chitin in the PM [12]. This would not only alter the chitin fibril structure but also could affect the interaction between PM components as well as the integrity and porosity of the structure. In fact, the PM-associated chitin deacetylase found in *Mamestra configurata* may create temporary, localized pores by disrupting chitin-PM protein interactions. The fact that knockdown of IsCDA did not affect overall PM organization suggests that either pre-existing or low levels of IsCDA are sufficient for normal PM existence or that its role is nonessential due to presence of other functionally relevant tick proteins. In fact, database searches indicated the existence of multiple *I. scapularis* gene products that display homology to the amino acid sequence of IsCDA (data not shown). 

Although the PM has been shown to affect transmission of certain arthropod-borne pathogens [28,29], the functions of the matrix in arthropod physiology or its role as an immune barrier to invading pathogens is largely unknown, even in model insects, such as *Drosophila* [30]. Although a protective role of the PM against *D. melanogaster* intestinal bacterial infection has recently been reported [30], its precise role in the persistence of bacterial pathogens, including *B. burgdorferi*, in ticks remains enigmatic. Similarly, the structure and functions of most PM proteins also remain unknown. While IsCDA contains a conserved enzymatic domain, substitutions of several amino acids are also noted in its predicted catalytic motif (Figure 3). This raises a strong possibility that IsCDA might not be enzymatically active; rather, it contributes to a supporting, nonessential role – such as strengthening the mechanical properties of chitinaceous structures, as postulated for other arthropod CDA-like proteins [24]. In fact, the latter speculation is supported by our current observation that IsCDA knockdown failed to affect PM occurrence or pathogen persistence, while treatment with IsCDA antibodies influenced *B. burgdorferi* levels in ticks, possibly affecting porosity (or permeability) of the PM. On the other hand, these antibodies could have also interfered with the functions of other unidentified proteins integral to normal PM formation or function via steric hindrance. Either way, such IsCDA antibody-mediated interruption predominantly affected borrelial persistence but did not influence levels of total gut bacteria, raising the intriguing possibility that the *I. scapularis* PM plays a selective role in controlling the levels of *B. burgdorferi* within the tick gut. Such species-specific effects of arthropod innate immune components have been contemplated earlier [31]. However, precisely how IsCDA antibodies modify the PM or help the survival of spirochetes remains to be elucidated. Lyme disease pathogens primarily reside in endoperitrophic luminal spaces and replicate there during tick feeding phases [7]. Therefore, antibody-mediated alteration of the molecular organization of the PM or its function or porosity could limit the ability of *B. burgdorferi* to effectively disseminate through the gut barrier, thus rendering the spirochetes more vulnerable to the hostile environment of the gut lumen and/or the innate immune attack mounted by gut epithelial cells. Further understanding the organization of the PM and its role in pathogen persistence will enrich our knowledge of tick-pathogen interactions and contribute to the development of novel preventions against vector-borne diseases.
